# Pre-attentive cortical processing of behaviorally perceptible spatial changes in older adults—a mismatch negativity study

**DOI:** 10.3389/fnins.2014.00146

**Published:** 2014-06-16

**Authors:** Claudia Freigang, Rudolf Rübsamen, Nicole Richter

**Affiliations:** ^1^Faculty of Bioscience, Pharmacy and Psychology, University of LeipzigLeipzig, Germany; ^2^IMPRS NeuroCom, Max Planck Institute for Human Cognitive and Brain SciencesLeipzig, Germany

**Keywords:** minimum audible angle, mismatch negativity, age-related, temporal processing, change detection

## Abstract

From behavioral studies it is known that auditory spatial resolution of azimuthal space declines over age. To date, it is not clear how age affects the respective sensory auditory processing at the pre-attentive level. Here we tested the hypothesis that pre-attentive processing of behaviorally perceptible spatial changes is preserved in older adults. An EEG-study was performed in older adults (65–82 years of age) and a mismatch negativity (MMN) paradigm employed. Sequences of frequent standard stimuli of defined azimuthal positions were presented together with rarely occurring deviants shifted by 10° or 20° to the left or to the right of the standard. Standard positions were at +5° (central condition) from the midsagittal plane and at 65° in both lateral hemifields (±65°; lateral condition). The results suggest an effect of laterality on the pre-attentive change processing of spatial deviations in older adults: While for the central conditions deviants close to MAA threshold (i.e., 10°) yielded discernable MMNs, for lateral positions the respective MMN responses were only elicited by spatial deviations of 20° toward the midline (i.e., ±45°). Furthermore, MMN amplitudes were found to be insensitive to the magnitude of deviation (10°, 20°), which is contrary to recent studies with young adults (Bennemann et al., 2013) and hints to a deteriorated pre-attentive encoding of sound sources in older adults. The discrepancy between behavioral MAA data and present results are discussed with respect to the possibility that under the condition of active stimulus processing older adults might benefit from recruiting additional attentional top-down processes to detect small magnitudes of spatial deviations even within the lateral acoustic field.

## Introduction

In natural environments we are often confronted with a multitude of sound sources typically producing overlapping sound mixtures. Still, the auditory system is able to single out coherent acoustic objects. Through auditory processing, subjects are able to identify and focus on individual sound sources when confronted with complex acoustic scenarios, a process termed *auditory scene analysis* (ASA) (Bregman, 1990; Shamma and Micheyl, 2010). While the mechanisms underlying ASA are not fully understood, previous studies suggested pitch, timbre and location of sound stimuli as the most prevalent cues in ASA processing (e.g., Shamma and Micheyl, 2010). Localization of sound sources in the horizontal plane are based on the processing of interaural time and level differences (ITD; ILD) and of monaural spectral cues the latter being the result of signal filtering by the upper body, head, and pinnae (Middlebrooks and Green, 1991; Blauert, 1997). Such calculation of positional information by the central auditory system is necessary, since the respective information is not directly encoded on the cochlear basilar membrane. At the level of cortical auditory areas, the information on horizontal sound source positions is thought to be based on a population rate code (Makous and Middlebrooks, 1990; Stecker and Middlebrooks, 2003; Werner-Reiss and Groh, 2008; Salminen et al., 2012). Binaurally activated neuron populations in both cortical hemispheres are assumed to code for positions in either hemifield by their relative level of activation (hemispheric channel model, e.g., Magezi and Krumbholz, 2010, for review see McAlpine, 2005).

Many studies have shown that older adults are less accurate in localizing sound sources compared to young adults (*location identification tasks:* Abel and Hay, 1996; Abel et al., 2000; *detection tasks:* Cranford et al., 1993; *pointing tasks:* Dobreva et al., 2011; Neher et al., 2011; Freigang et al., 2014, review: Eddins and Hall, 2010), plus, there are a number of reports on a strong age-related decline in spatial discrimination as indicated by elevated Minimum Audible Angle (MAA) thresholds (Häusler et al., 1983; Chandler and Grantham, 1991; Freigang et al., 2014). The MAA is used as the measure of the smallest angular distance between two neighboring sound sources that can be detected correctly (Mills, 1958). It has been proposed that reduced sensitivity to location cues is due to both reduced peripheral hearing (Corso, 1971; Häusler et al., 1983; Abel and Hay, 1996;Cruickshanks et al., 1998) and impaired central auditory processing (CHABA, 1988; Chandler and Grantham, 1991; Humes, 1996; Noble et al., 1997; Dobreva et al., 2011; Neher et al., 2011). Others pointed to a possible contribution of age related changes in cognitive processes. For example, Bertoli et al. (2002) and Alain et al. (2004) reported that focusing attention on a gap detection task may help aged subjects to partly overcome degraded sensory processing. To date, it is not clear to what degree each of these factors contribute to the age-related decline in localization performance, since no data is available specifically focusing on physiological processing of auditory space information.

A sensitive tool to examine pre-attentive sensory processing at the level of the auditory cortex is the evaluation of auditory event-related potential (ERP) Mismatch Negativity (MMN, review: Näätänen et al., 1978). It is hypothesized that the MMN serves as an automatic process to alert the system to deviations in the unattended acoustic environment (Sams et al., 1985; Schröger, 1998; Winkler and Czigler, 1998). MMN has been used in many studies to assess the resolution of acoustic feature processing (e.g., frequency, duration, gap detection) including spatial acuity (Deouell et al., 2006; Pakarinen et al., 2007; Vaitulevich and Shestopalova, 2010; Bennemann et al., 2013). Deouell et al. (2006) reported for young adults MMNs elicitation for spatial signal separation of 10 degrees within the frontal acoustic field. For the mid-lateral (65°) and far-lateral (95°) positions, MMNs were elicited for spatial separation of 5° and 15°, respectively (Bennemann et al., 2013). These MMN data are consistent with behaviorally assessed human localization abilities (Blauert, 1997). Cortical generators for MMN were found bilaterally in the primary and secondary auditory cortices in the superior temporal gyri of the temporal lobes as well as in frontal, parietal, and supratemporal cortical sites (Giard et al., 1995; Kropotov et al., 1995; Picton et al., 2000). The MMNs are thought to have different generators activated in a feature-specific fashion by frequency, duration, or location cues (Paavilainen et al., 1991; Alho, 1995; Picton et al., 2000; Deouell et al., 2006). Relating to the present study, MMN has been reliably elicited in experiments probing location differences by either varying (i) ITDs under headphone conditions (Schröger and Wolff, 1996; Schröger, 1996; Pakarinen et al., 2007), (ii) real spatial disparities under free field conditions (Paavilainen et al., 1989; Nager et al., 2003; Tata and Ward, 2005; Deouell et al., 2006; Richter et al., 2009; Grimm et al., 2012; Bennemann et al., 2013) and (iii) using headphone stimulation but employing head-related transfer functions conditions (Sonnadara et al., 2006). Such MMN components are often followed by the ERP P3a (occurring 200–350 ms post stimulus onset), which is thought to indicate an involuntary switch in attention toward the deviant sound (Picton et al., 2000).

Previous studies showed that the MMN amplitude is reduced in elderly subjects, possibly due to an age-dependent decline in pre-attentive automatic central auditory processing (frequency: Czigler et al., 1992; Schroeder et al., 1995; Alain and Woods, 1999; duration: Woods, 1992; Karayanidis et al., 1995; Pekkonen et al., 1996; Bertoli et al., 2002; Ruzzoli et al., 2012; gap detection: Alain et al., 2004). However, the underlying mechanisms—mostly considered in relation to frequency detection—are still controversially discussed. On the one hand, the findings were interpreted as *impairment* in maintenance of the sensory memory trace in older adults. On the other hand, the reduced MMNs were considered as an indication for an impairment of the encoding of sensory information (Czigler et al., 1992; Gunter et al., 1996; Pekkonen, 2000; Cooper et al., 2006). Moreover, there are studies (on frequency discrimination) that did not report any age effect at all (Schroeder et al., 1995; Amenedo and Diaz, 1998).

The present study focuses on the question, whether the age-related localization acuity acquired behaviorally from the MAA (Freigang et al., 2014) is already reflected at a pre-attentive level indicated by the MMN, i.e., at an early level of cortical auditory processing and mostly independent of attentional top-down modulations. For this, previously reported (attentive) behavioral MAA thresholds were used as a basis to perform an MMN experiment using a passive (unattended) stimulation condition. Deviants with two spatial disparities were used: 10°, and 20° i.e., on the one hand near to and on the other hand above pericentral MAA thresholds. Furthermore, in addition to the pericentral (5°) also the lateral (65°) acoustic field was explored for deviants shifting both toward the midline and toward the sides. This specific stimulus design enables the evaluation of the acuity in pre-attentive cortical representation of auditory spatial information considering both, stimulus laterality (pericentral vs. lateral) and the direction of spatial change (toward the midline vs. toward the sides; Richter et al., 2009; Bennemann et al., 2013).

If in older adults behaviorally manifested localization acuity (MAA) corresponds to the automatic, pre-attentive cortical encoding (MMN), the latter should yield responses for near- and above-threshold spatial deviations, in particular within the central acoustic field. If, however, the pre-attentive cortical representation of acoustic space is blurred in older adults, then (i) no or only above-threshold deviations are expected to elicit MMN responses and/or (ii) no magnitude effect with respect to the size of the spatial deviations should be found.

## Materials and methods

### Subjects

Fifteen older adults (65–82 years-of-age [66.8 ± 4.74 years], 7 women) participated in the MMN experiment. Subjects signed an informed consent form and received a compensation for expenses. The study was approved by the ethics committee of the University of Leipzig and is in agreement with the revised Declaration of Helsinki. All subjects performed the Edinburgh Handedness Inventory (Oldfield, 1971), were screened for cognitive deficits with the Mini-Mental State Examination [MMSE] (Folstein et al., 1975), and underwent audiometric testing. All subjects scored 27–30 points in the MMSE identifying them as non-conspicuous. The subjects were also screened for hearing loss and only subjects were included, whose pure-tone thresholds at 500 Hz, 1 kHz, and 2 kHz were on average ≤25 dB HL (hearing level). Data from two subjects had to be excluded from the EEG analysis because of multiple movements-induced disruptions of the recordings.

### Experimental setup and EEG recordings

Audiometric testing was conducted in an anechoic, sound attenuated test booth (Industrial Acoustics Company, IAC Type 403 A, Niederkrüchten, Germany). Pure-tone thresholds were examined via headphones (Beyerdynamics, DT 770 Pro). Sounds were generated with a sampling rate of 25 kHz by the real-time processor RP2.1 [Tucker Davis Technologies (TDT), System III], and transmitted to headphones via a headphone power amplifier (TDT, HB7). Stimulus generation and hearing threshold acquisition were controlled by MATLAB (version 6.3, The MathWorks Inc., Natick, USA) (Biedermann et al., 2008).

**Figure 1:**
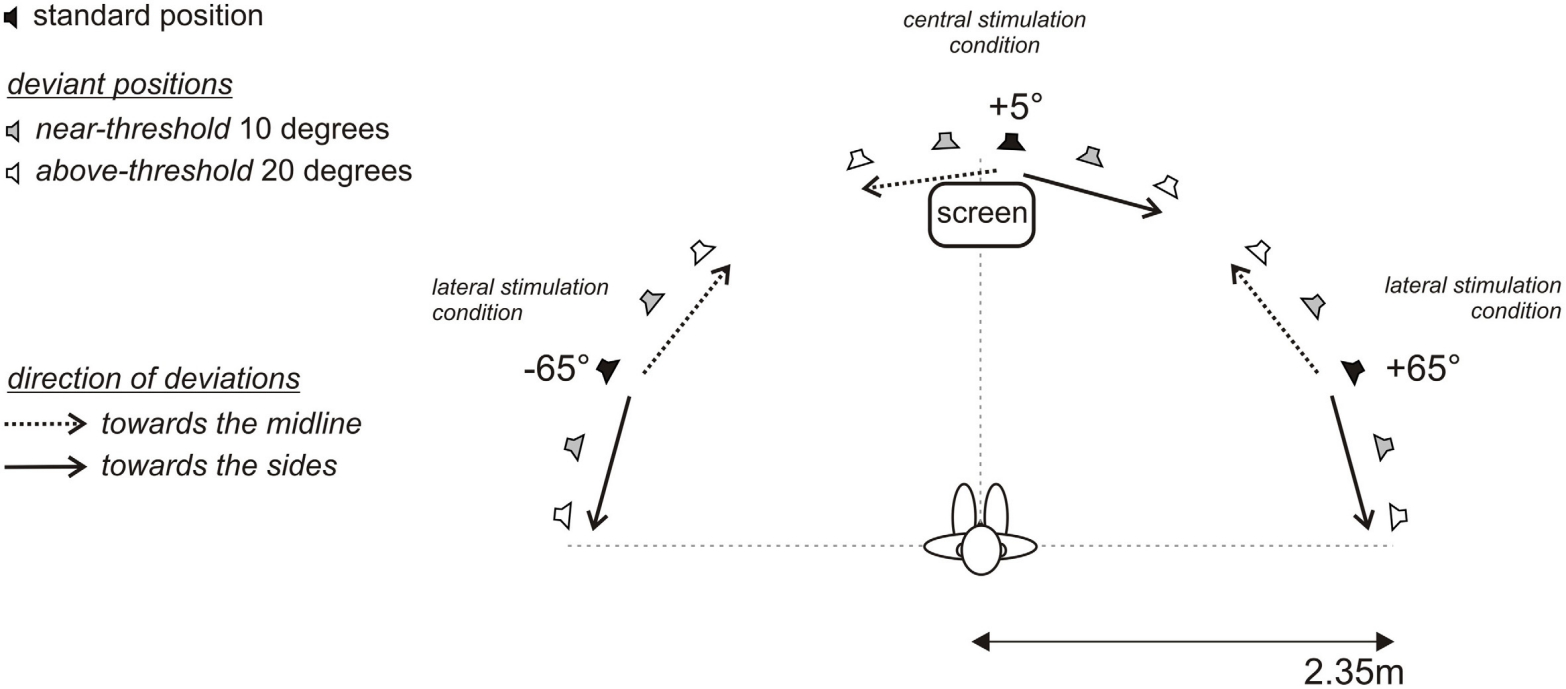
**Free-field setup with 33 loudspeakers in a semicircular array positioned between 85° to the left (−) and 85° to the right (+)**. Acoustic targets of defined lateral positions were established by activation of a single loudspeaker or crossfading the inputs to two neighboring speakers. Subjects were seated in the center of the semicircle with the head oriented straight and looking at a fixation cross at 0° (vertical dashed line); the interaural axis extended to the positions −90° and +90°, respectively (horizontal dashed line). In three blocks standard signals were presented at −65°, +5°, and +65°, (black loudspeaker symbols), and respective deviant signals were spatially displaced by 10° (gray loudspeaker symbols) and 20° (white loudspeaker symbols) toward the midline (dotted arrow) and the sides (solid arrow). Subjects were instructed to ignore the acoustic stimulation and to attend a muted movie with subtitles presented on a screen frontally at 0°.

The MMN experiment were conducted in an anechoic, sound attenuated free-field laboratory (45 m^2^, IAC, Figure 1). Thirty-three broad-band loudspeakers (Visaton, FRS8 4 Ohm, Haan, Germany) mounted in an azimuthal, semicircular array at ear level were used for sound stimulation. A comfortable, fixed chair was positioned in the middle of the semicircle at a distance of 2.35 m from the loudspeakers, such that subjects were aligned straight ahead to the central speaker at 0°. The speaker array covered an azimuthal plane from 85° to the left to 85° to the right (−85°; +85°). The angular distance between two speaker membranes was 4.3° as measured between the centers of the speaker membranes. In the experiments, a minimal distance between two sound sources of 2.1° was achieved by crossfading the signals of two neighboring speakers. That is, two speakers were simultaneously active and the in-between speaker position was generated by varying the relative sound levels of each speaker. Speakers were calibrated individually (for details on the calibration procedure see Schmiedchen et al., 2012). Speakers were hidden behind acoustically transparent gauze, so the participants were unable to make use of visual landmarks during the experiments. The passive MMN experiment was conducted at a low light level. A movie was played from a screen positioned in front at 0° of the subject slightly below the speaker membrane. During the experiment the participants were monitored by an infrared camera. MATLAB (version R2007b) was used to control stimulus presentation and data acquisition. Acoustic stimuli were digitally generated at a sampling rate of 25 kHz using RPvdsEx (Real Time processor visual design studio, TDT) and delivered to two multi-channel signal processors (RX8, TDT System3).

The EEG was recorded with a 64-channel Ag/AgCl active electrode system (actiCAP, Brain Products) according to the international 10-10 system (American Electroencephalographic Society, 1994). Fifty-eight channels were used to record neuronal activity from the scalp. Four electrodes used to record vertical and horizontal electrooculograms (EOG), so subsequently epochs with massive eye movements could be disregarded in the analysis. Two additional electrodes were used to pick up signals at the left and right mastoid sites. The reference electrode was placed on the tip of the nose and the ground electrode at position Fpz. Impedances were kept below 10 KOhm and recorded signals were online sampled with 500 Hz and band-pass filtered between 0.1 and 100 Hz.

### Acoustic stimuli

Stimuli were low-pass filtered Gaussian noise bursts (300–1200 Hz), 250 ms in duration with 10 ms cos^2^ ramps (Richter et al., 2009; Bennemann et al., 2013). The interstimulus interval (ISI) varied randomly between 350 and 450 ms in increments of 10 ms (average ISI = 400 ms). A level roving of ±2.5 dB was applied in randomized 1 dB steps to prevent MMN-effects by loudness cues due to minute differences in the position or orientation of the loudspeakers.

### Experimental procedure and data acquisition

#### Measurement of individual hearing thresholds

Prior to recording EEG, the subjects' individual hearing thresholds were acquired using a yes-no detection criterion combined with a simple staircase paradigm. For this, the low-pass noise signals were presented from the frontal position at 0° (see Figure 1) with an initial intensity of 63 dB SPL. Subjects were instructed to press the left button on a response box to indicate when they detected a sound (*yes*-response) and the right button when they did not detect the sound (*no*-response). Intensity was decreased by 2.5 dB for each *yes*-response and increased by 2.5 dB for each *no*-response. The stimulus level at the fourth no/yes response switch was taken as the detection threshold. In the EEG experiment the acoustic stimuli were presented at 40 dB SL.

#### EEG experiment

A passive oddball paradigm was used. Participants were watching a silent, subtitled movie and were instructed to entirely direct their attention on the movie and to ignore the sounds. The recordings were organized in three main and twelve control blocks. Each main block consisted of 1600 standards and 400 deviants with the standards presented from 65° in the left hemifield (−65°), from 5° to the right of the median plane (+5°), and from 65° in the right hemifield (+65°); standard probability was 0.8 (Figure 1). Deviants were presented each with a probability of 0.05. During the recordings a multiple deviant paradigm was applied (Pettigrew et al., 2004; Deouell et al., 2006; Sambeth et al., 2009; Bennemann et al., 2013). Deviants were shifted by 10° or 20° away from the respective standard positions, either toward the midline or toward the side, i.e., for the +5° central condition the deviants were at −15°, −5°, +15°, +25° and for the ±65° standard positions the deviants were at ±85°, ±75°, ±55°, ±45°, respectively. A sparse presentation paradigm was used, with at least three standards between two subsequent deviants. Additionally, twelve control blocks were recorded, where stimuli, previously used as deviants, were presented as standards with a probability of 0.8. Each control block consisted of 100 standards (deviants as standards | main block) and 20 deviants (standard as deviant | main block). The standards from the control blocks and respective deviants from the main blocks were used in the analyses to determine MMN responses caused by changes in spatial position rather than by different representations of physically different stimuli (Kujala et al., 2007).

### Data analysis

EEG data were preprocessed offline and analyzed by using the Matlab toolbox EEGLAB (version 10.0.0.0b; Delorme and Makeig, 2004; http://sccn.ucsd.edu/eeglab/). Continuous recordings were FIR band-pass filtered between 1 and 20 Hz. Subsequently, data were segmented for the different deviant and standard conditions by extracting 600 ms epochs which comprise the period 100 ms before stimulus onset (baseline) and 500 ms epochs after stimulus onset. Epochs were baseline-corrected by referencing the channel means to the respective baseline and linear trends were removed from each epoch by applying drift correction. Epochs with amplitudes exceeding ±90 μV were excluded from further analysis and an average of 56 epochs out of 100 was kept per person. Epochs of all main and control blocks were averaged individually for each condition (*central*, *lateral*) and respective standard and deviant positions (−15°, −5°, +15°, +25°, and ±85°, ±75°, ±55°, ±45°). Difference waves (DW) were computed by subtracting the ERPs of deviants presented as standards (control stimuli) in control blocks from the ERP of deviants presented in the main block, i.e., DW_deviant_ = ERP_deviant_main block_ − ERP_deviant as standard_control block_.

Respective grand averages were computed separately from the averages of individual subjects. To increase the signal-to-noise ratio (SNR) for MMN, DW were additionally re-referenced to the mastoids (Kujala et al., 2007). To further increase SNR for the *lateral* blocks, we collapsed the ERP data elicited by deviants presented at ±45°, ±55°, ±75°, and ±85° across hemifields.

To test for statistical significance of MMN and P3a signals, mean MMN and mean P3a amplitudes were measured for each subject within a window of ±10 ms around the peaks of the corresponding grand averaged responses. Mean MMN and mean P3a amplitudes were tested against zero with a one-sample, two-tailed Student's *t*-test. MMN and P3a signals that failed to reject the null hypothesis were excluded from further analyses. Following this procedure, individual MMN and P3a amplitudes were calculated as the mean within a ±10 ms time window around the individual MMN and P3a latencies measured at the peak amplitude in the respective time windows (100–250 ms for MMN, 200–350 ms for P3a post stimulus onset).

For the analysis of individual MMN amplitudes and latencies for *central* and *lateral* stimulus conditions the electrodes Fz was preselected. Furthermore, respective statistical differences were evaluated by a Two-Way rm-ANOVA including the factors “*direction of deviation*” (toward the midline, toward the side) and factor “*magnitude of deviation*” (10°, 20°). The analyses of inter-hemispheric differences in MMN amplitude was based on a spatial average of selected left (F2, F4, F6, FC2, FC4, FC6) and right (F1, F3, F5, FC1, FC3, FC5) electrode sites. Left- and right-hemispheric mean amplitude distributions of respective MMN components were tested against zero with a one-sample, two tailed Student's *t*-test. The effects of sound source laterality (central +5° vs. lateral ±65°) on MMN amplitude and latency was tested by *post-hoc* paired comparison *t*-tests. For this, we selected the MMN responses elicited by a spatial deviation of 20° toward the midline (at −15° for the central block and ±45° for the lateral block) evoked at electrode *Fz*. Voltage topographies of MMN and P3a components were analyzed separately in the respective components' time windows using the open source toolbox *sphspline*, which is based on spherical interpolation (https://github.com/widmann/sphspline; Perrin et al., 1989). The Greenhouse–Geisser correction was applied. All selected comparisons were made by using Bonferroni-corrected paired *t*-tests.

## Results

**Figure 2:**
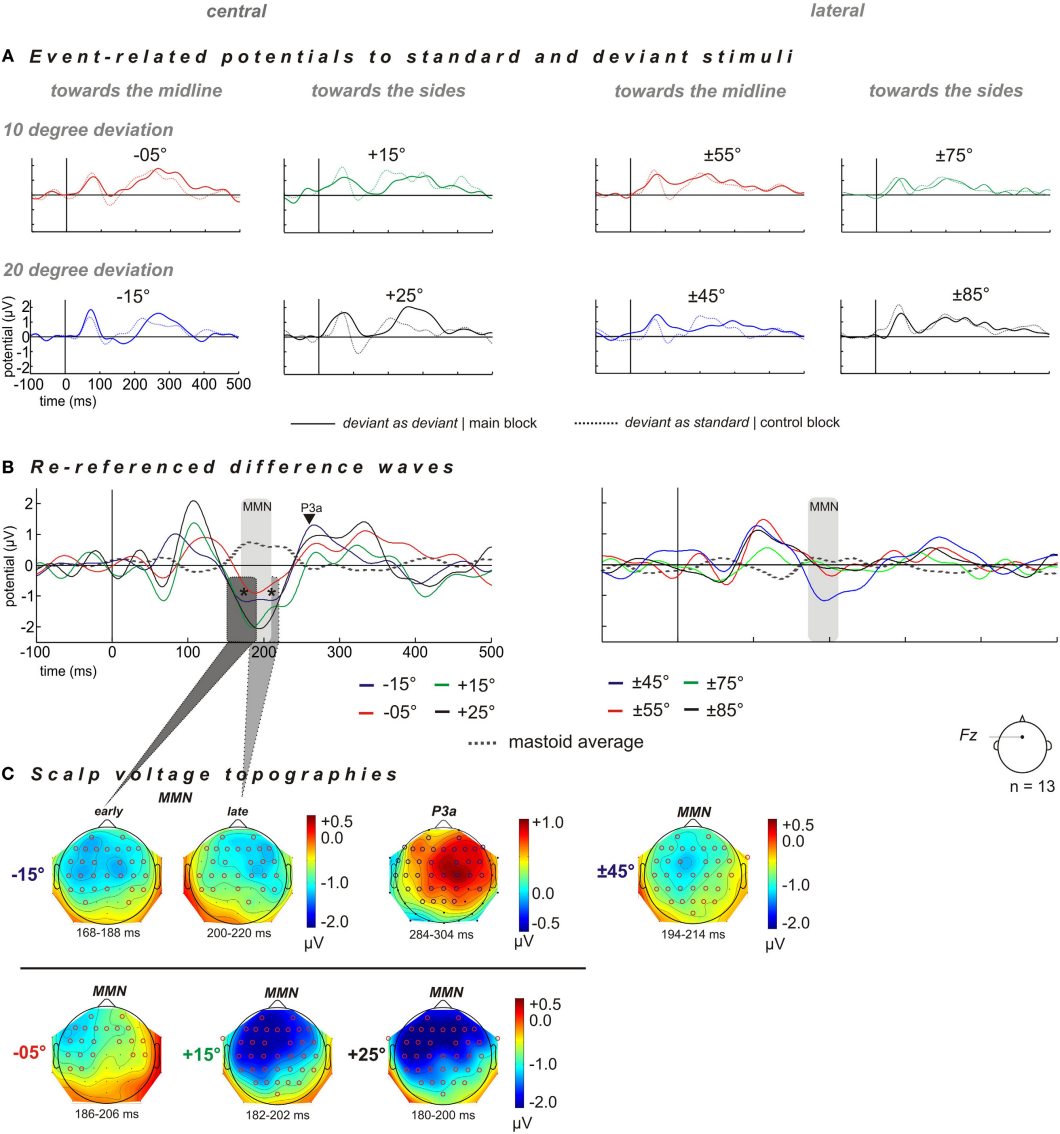
**Grand averaged ERP shown for scalp electrode Fz and distribution of deviant-related activity obtained for *central* (left column) and *lateral* (right column) reference positions**. The spatial deviations relative to the standard positions are color-coded: blue −20° and red −10° for spatial displacements *toward the midline*, and green +10° and black +20° for spatial displacements *toward the sides.* Central stimulation: deviant positions at −15° (blue), −05° (red), +15° (green), and +25° (black). Lateral stimulation: deviant positions at ±45° (blue), ±55° (red), ±75° (green), and ±85° (black). **(A)** Grand averaged ERP to “deviant as deviant” (solid line) and “deviant as standard” (dashed line). **(B)** “Deviant as deviant”–“deviant as standard” ERP difference waves (solid lines) re-referenced to ERP signal obtained at mastoid electrodes (dashed gray lines). For the condition “central” all deviants elicited sizeable MMN responses. Deviant position −15° evoked a two-tailed MMN component, i.e., early and late MMN components within a time window of 168–188 ms and 200–220 ms, respectively (indicated by asterisks), followed by a P3a component in the time window of 285–205 ms after stimulus onset. For the condition “lateral,” only the deviant position at ±45° elicited sizeable MMN responses. **(C)** Scalp voltage topographies of the MMN and P3a amplitudes for respective deviant positions, significant electrodes sites were shown as red and blue circles (one-sample, one-sided *t*-test, *p* < 0.05) *n* = 13.

ERPs were elicited for deviants and the corresponding control stimuli (“deviant as standard”) at each of eight deviant positions (−15°, −5°, +15°, +25°, ±45°, ±55°, ±75°, and ±85°; cf. Figure 2) and respective MMN amplitudes are listed in Table 1. Potential topographies of DWs (“deviant” – “deviant as standard”) within the MMN latency time window of significantly evoked MMNs show broadly distributed negative deflections over frontocentral scalp sites, with the polarity inverting at mastoid sites (Figures 2B,C). The scalp voltage topographies point to putative MMN generators within both cortices, including frontal, supra-temporal, and parietal areas.

**Table 1:** **Mean MMN amplitudes and latencies for the 8 deviants measured at electrode Fz**.

**Deviant position (direction of deviation)**	**Mean amplitudes^a^ (±SEM)/μV**	**MMN latencies^b^ (±SEM)/ms**
−15° (toward the midline)	early −1.18 (0.46)^*^	178.46 (3.84)
	late −1.13 (0.45)^*^	210.00 (4.15)
−5° (toward the midline)	−0.89 (0.29)^*^	197.08 (6.40)
+15° (toward the sides)	−1.98 (0.46)^*^	192.20 (6.22)
+25° (toward the sides)	−2.04 (0.46)^*^	198.61 (4.16)
±45° (toward the midline)	−1.15 (0.49)^*^	203.80 (6.65)
±55° (toward the midline)	−0.36 (0.24)	−
±75° (toward the sides)	−0.18 (0.27)	−
±85° (toward the sides)	−0.18 (0.37)	−

Mean amplitudes were calculated within ±10 ms around the peak MMN that occurred 150–250 ms after stimulus onset from grand average re-referenced ERPs. A one-sample two-sided t-test (df = 12) was calculated to test if MMN amplitudes within the time window form a distribution with the mean zero. Significant values are indicated by asterisks, with

* p ≤ 0.05.

a MMN amplitudes were obtained within the ±10 ms time window around the latency of the MMN peak in the re-referenced grand averages.

b Individual MMN latencies measured from individual difference waves at the MMN peak amplitude.

### Central stimulus condition (+5° standard position)

Grand averaged ERPs for deviants show a characteristic negative deflection compared to the corresponding ERPs for control stimuli in the MMN time window 140–220 ms post stimulus onset at electrode *Fz* (Figures 2A,C). Thus, each deviant stimulus reliably elicited an MMN signal within a latency window of 140–220 ms [−15°: *t*_(12)_ = −6.1387, *p* < 0.001; −5°: *t*_(12)_ = −6.6611, *p* < 0.001; +15°: *t*_(12)_ = −7.1795, *p* < 0.001; +25°: *t*_(12)_ = −7.7759, *p* < 0.001; Table 1, Figure 2]. Deviants presented at −15° evoked an MMN followed by a P3a at around 300 ms [295 (±11 [SEM]) ms] with an amplitude of 1.24 (±0.5 [SEM]) μV [*t*_(12)_ = 2.5470, *p* = 0.0256], while all the other deviant positions (−5°, +15°, +25°, ±45°, ±55°, ±75°, and ±85°) failed to elicit significant P3a-amplitudes (all *p* > 0.05).

The rm-ANOVA revealed a significant effect of factor “direction of deviation” [*F*_(1, 12)_ = 7.0792, *p* = 0.0208] with larger MMN amplitudes evoked by spatial deviations toward the sides than toward the midline [MMN_side_: −1.96 (±0.33) μV] > MMN_midline_: −1.01 (±0.2) μV, *t*_(12)_ = 3.4387, *p* = 0.0049. Furthermore, no significant main effect of factor “magnitude of deviation” [*F*_(1, 12)_ = 0.1943, *p* = 0.6672] or significant interactions were found [“magnitude of deviation” × “direction of deviation”: *F*_(1, 12)_ = 0.0618, *p* = 0.8078].

The MMN latencies were not affected by factors “direction of deviation” [*F*_(1, 12)_ = 0.7060, *p* = 0.4172] and “magnitude of deviation” [*F*_(1, 12)_ = 0.9329, *p* = 0.3532]. Also no significant interactions were found [“magnitude of deviation” × “direction of deviation”: *F*_(1, 12)_ = 0.9329, *p* = 0.3532].

### Early and late MMN components at deviant position −15°

The 20° deviation toward the midline elicited two negative peaks referred to as early and late MMN components (indicated by asterisks in Figure 2C). The early MMN component peaked at 178 (±4) ms with a mean amplitude of −1.16(±1.6) μV [*t*_(12)_ = −16.3762, *p* < 0.001]. The late MMN component had its maximum at 210 (±4) ms with a mean amplitude of −1.10 (±1.5) μV [*t*_(12)_ = 19.543, *p* < 0.001]. When comparing the topographies, the early MMN component yielded a uniform left- and right-hemispheric distribution unlike the late component, which showed a more rightward lateralization (cf. Figure 2C). However, the inter-hemispheric comparisons of left-and right hemispheric MMN amplitudes within the time windows of the early and late MMN components did not reveal a significant difference for either of the two [early MMN: left vs. right hemisphere: *t*_(12)_ = −0.8104, *p* = 0.4335; late MMN: left vs. right hemisphere *t*_(12)_ = 1.2338, *p* = 0.2409].

### Lateral stimulus condition (standard at ±65°)

For the lateral stimulus condition MMN signals elicited by deviants at ±55°, ±75°, and ±85° failed to reach significance levels (all *p* > 0.05). The MMN amplitudes only yielded significance for spatial deviations toward the midline at ±45° [*t*_(12)_ = −2.337, *p* = 0.0376] peaking at 203 (±7) ms.

### No effect of sound source laterality on MMN amplitude and latency

To evaluate the putative effect of *sound source laterality* on the MMN, the responses elicited by 20° spatial deviations toward the midline at central and lateral reference position were evaluated. The comparison yielded no significant effect of sound source laterality [MMN amplitude: central vs. lateral: *t*_(12)_ = −0.1151, *p* = 0.9103; MMN latency: central vs. lateral: *t*_(12)_ = −1.7351, *p* = 0.1083].

## Discussion

The present study examined early sensory auditory processing to changes in sound location in older adults by recording MMN. Previous studies showed that spatial auditory acuity—evaluated by measuring the MAA—is declined in older adults (Freigang et al., 2014). This led to the question, whether this decline in performance is also reflected in early neuronal processes linked to auditory discrimination. To get a better understanding of this issue, we measured the MMN in older adults to 10° and 20° spatial deviations, which corresponds to behaviorally acquired near and above MAA threshold values within the central field. In the present study, reliable MMNs were recorded for 10° and 20° spatial deviations from the +5° central position and for 20° deviations (i.e., at ±45°) from the lateral ±65° standard positions.

### Decreased fine-tuning in the pre-attentive processing of pericentral sound sources

For frontal positions, MMNs were found for corresponding near-threshold and above-threshold deviants (Freigang et al., 2014) for both directions of deviation, i.e., toward the midline and toward the sides. Thus, considering that the MMN response is informative about an intact automatic change detection process, which relies on an adequate neuronal integration of the sensory input at subcortical and cortical level, it can be concluded that spatial changes measured behaviorally were also pre-attentively detected in older adults.

However, while in recent studies examining young adults the MMN amplitudes increases with increasing magnitude of spatial deviation (Deouell et al., 2006; Bennemann et al., 2013), here for both magnitudes of deviation (10°, 20°) equal MMN amplitudes were found. This finding implies a loss of at least 10° in gradual coding of auditory space representation at a preattentive level (Deouell et al., 2006; Bennemann et al., 2013). Notably, Deouell and colleagues used 50 ms long spectrally rich tones (fundamental 500 Hz, and three partials [1000, 1500, 2000 Hz]) which enabled the subjects to use ITD cues (based on the 500–1500 Hz-partials) as well as ILD cues (2000 Hz-partials). The presently used low-frequency noise bursts (300–1200 Hz) with a length of 250 ms predominantly provided ITD cues for sound source processing (Blauert, 1997). Given these differences, no definite conclusion can be drawn upon whether the absence of an increase in MMN amplitude with increasing magnitude of deviation can directly be related to differences in age or to the differences in stimulus design. Interestingly, in an MMN study on sound localization by Paavilainen et al. (1989), the same effects of spatial deviations on MMN were reported for both, low- and high-frequency sounds. Furthermore, they found MMN latencies to be shorter with increasing spatial deviation, an effect even more prominent for low- than for high-frequency sounds.

In the study of Deouell et al. (2006) stimuli had a duration of 50 ms, while in the present study the duration of the noise bursts were 250 ms. The longer stimuli were motivated by previous MMN studies suggesting a relative long “temporal window of integration” for auditory events (e.g., Tervaniemi et al., 1994; Winkler et al., 1998; Yabe et al., 1998) requiring stimuli of at least 150–300 ms in duration to achieve full integration of all stimulus-specific acoustic information. Also, a behavioral study by Grantham (1995) on the ability to detect dynamic interaural cues suggested that signal durations of 150–300 ms are necessary to warrant the lowest possible thresholds in binaural discrimination. Considering these findings, we would like to argue that the present mode of acoustic stimulation ensures the full modulating effects of spatial deviation on MMN. Still, further test are needed to clarify the postulated relation between age and pre-attentive encoding of sound sources and these studies will have to include a control group with young adults to enable a direct comparison.

### Decreased neural resolution of space within the lateral acoustic field

For the lateral ±65° positions, valid MMN responses were obtained for 20° deviations toward the midline (±45°) but not for the same magnitude of deviation toward the sides. These findings suggest that spatial changes toward the sides of about 16°—verified to be distinguishable by older adults in behavioral experiments (Freigang et al., 2014)—were pre-attentively unrecognized by the MMN system. In young adults, spatial changes of 17° either toward the midline or to the sides within the lateral acoustic field were shown to elicit valid MMN responses (Richter et al., 2009). More specifically, in a study on young adults by Bennemann et al. (2013) the same laterality of 65° was explored and MMN responses were obtained for lateral spatial deviations of 5°, 10°, and 15° toward the sides (i.e., respective deviants at 70°, 75°, and 80°). Furthermore, in the same subjects monotonously increased MMNs were found with larger spatial deviations which led to the ascertainment of at least 5° resolution of neuronal pre-attentive sound source discrimination at such lateral positions. Since—as presently shown—in older adults neither lateral spatial displacements of 10° or 20° toward the sides elicited MMNs, we suspect that the preattentive fine-grained encoding of far-lateral sound sources by the MMN system deteriorates with age. Localization of low-frequency sounds is predominantly based on the processing in ITD (e.g., Middlebrooks and Green, 1991) a finding which might relate to results of earlier studies showing impaired processing of ITD information in older adults (Kirikae et al., 1963; Herman et al., 1977; Strouse et al., 1998; Babkoff et al., 2002). Also, it was shown that in elderly adults the fidelity in encoding temporal information was generally declined (Ross et al., 2007, 2010; Ross, 2008; Ruggles et al., 2011, 2012), which is in agreement with decelerated temporal processing mechanisms (Pichora-Fuller and Schneider, 1991; Frisina and Frisina, 1997; Schneider and Hamstra, 1999; Lister and Roberts, 2005; Freigang et al., 2011). These findings are in line with the notion of a blurred representation of sound sources in older results, particularly for low-frequency sounds.

For the ±65° standard positions, MMNs were elicited by deviants displaced by 20° toward the midline possibly pointing to a specific relation between the position at which a “novelty” occurs and a given reference position. Considering the fact that changes in interaural acoustic cues decrease with increasing lateralities up to 90° (Blauert, 1997; Moore, 1997), the presently tested 20-degree-deviation toward the side (re 65° standard position) resulted in smaller ITD-changes than the same spatial deviation toward the midline. This indicates that the later ITD-changes reached values that were pre-attentively detected by the MMN system.

Still, there is a discrepancy between the MMN data acquired here and previously reported MAA data (Freigang et al., 2014), which might suggest a role of spatial selective attention mechanisms in improving auditory discrimination of adjacent sound sources especially in the lateral acoustic space (for further details see Bennemann et al., 2013).

Previous studies reported that older people benefit from an active attentional focusing possibly compensating for the age-related decline in automatic establishment of memory traces (Bertoli et al., 2002; Alain et al., 2004). Alain et al. (2004) recorded active and passive MMNs in a gap detection task and found that when attention was directed away from the auditory modality, or explicitly focused on a visual task, the physiological response to an near-threshold deviant was absent in older adults. In young adults, in contrast, auditory near-threshold deviants elicited MMNs despite attention being focused on a visual task. The present findings are in agreement with the notion of a general age-effect on the preattentive processing of sound properties (Alain and Woods, 1999; Bertoli et al., 2002; Alain et al., 2004) and refer to its specific importance for auditory space processing (Freigang et al., 2014).

### Effect of direction of deviation on MMN within the pericentral space

For the +5° standard position, larger MMN amplitudes were elicited by positional changes toward the sides than toward and across the midline. Noteworthy, the respective deviants at +15° and +25° were within the same hemifield, while the deviants at −5° and −10° were in the opposite hemifield. The larger MMN amplitudes might relate to stronger activation of a defined neuron population and/or activation of a larger neuron population. Interestingly, the respective MNN differences are contradictory to predictions emanating from the “opponent-channel coding” hypothesis (also referred to as hemifield code), the prevailing model for cortical representation of acoustic space (Stecker and Middlebrooks, 2003; Werner-Reiss and Groh, 2008; Magezi and Krumbholz, 2010; Salminen et al., 2012; Briley et al., 2013). The model proposes for each cortical hemisphere neuronal populations tuned to the respective contralateral acoustic hemifield exhibiting activation profiles with maxima for lateral sound source positions and steeply decreasing slopes toward central positions. Still, behavioral studies suggest that both hemifield channels overlap in an area of about 30° in the frontal acoustic field (Boehnke and Phillips, 1999; Phillips, 2008). According to this model, scalp recordings from vertex sites should yield higher cortical activity levels for sounds (i) emanating from lateral compared to central positions (*EEG:* Magezi and Krumbholz, 2010; Briley et al., 2013) and (ii) originating from opposite hemifields (*MEG*: Salminen et al., 2012). Previous studies testing the opponent channel coding employed rather large angular distances (30°, 45° to 90°) and did not specifically explored frontal areas of overlapping hemifield channels. Since presently the positional changes fall into the 30° range of overlap of both hemifield channels, it is not possible to anticipate potential effects on the MMN.

Also, there is a possibility that for the central stimulation not only the factor “direction of deviation” might affect the MMN amplitude, but also the factor “crossing the midline.” For our experiments we chose the same standard position at +5° as Deouell and coworkers in an earlier study on young adults (Deouell et al., 2006). In this study the deviants were located at +15° (i.e., more lateral) and at −5° (i.e., to the front and crossing the midline). Unlike in the present study, in young adults both deviants elicited equal MMN responses. Same as in the study by Deouell and coworkers, the MMN had a two-peak structure (termed early and late MMN components) followed by a P3a component for deviants presented at −15° (Deouell et al., 2006). Presently, the occurrence of the deviant across the midline might have established the condition to evoke an involuntary attentional shift, which is what the P3a is thought to stand for (e.g., Horvath et al., 2008).

## Conclusion

The present study suggests that the pre-attentive processing of changes in spatial positions can be impaired in older adults despite the fact that the same spatial changes are behaviorally distinguishable. This particularly holds for lateral positions, while preattentive sound source processing is largely preserved for sources within the pericentral and mid-lateral acoustic fields. The constancy of MMN amplitudes for different magnitudes of spatial deviation suggests a decline in spatial resolution. We hypothesize that older adults might benefit from actively engaging top-down attentional processes to detect small magnitudes of spatial changes specifically in the lateral acoustic field.

### Conflict of interest statement

The authors declare that the research was conducted in the absence of any commercial or financial relationships that could be construed as a potential conflict of interest.
